# A Phase II, Randomized, Double-Blind, Double-Dummy, Active-Controlled Clinical Trial to Investigate the Efficacy and Safety of NW Low-Glu® in Patients Newly Diagnosed with Type 2 Diabetes Mellitus

**DOI:** 10.1155/2022/9176026

**Published:** 2022-09-27

**Authors:** Samir Assaad-Khalil, Nabil Elkafrawy, Mohsen Khaled, Omneya Mogeib, Hytham Badr, Ahmed Rashwan, Mahmoud Youssef, Khaled Eltamawy, Shahnaz Mohamed

**Affiliations:** ^1^Department of Internal Medicine, Unit of Diabetology, Lipidology & Metabolism Faculty of Medicine, Alexandria University, Alexandria, Egypt; ^2^Department of Internal Medicine, Unit of Endocrinology & Diabetes, Faculty of Medicine, Menoufia University, Shebin El Kom, Menoufia, Egypt; ^3^Egyptian National Institute of Diabetes and Endocrinology, Cairo, Egypt; ^4^Department of Endocrinology, National Research Center, Giza, Egypt; ^5^Unit of Critical Care Medicine, Fayoum General Hospital, Fayoum, Egypt; ^6^Department of Cardiovascular Medicine, Faculty of Medicine, Mansoura University, Mansoura, Egypt; ^7^Unit of Cardiology, Assiut General Hospital, Assiut, Egypt; ^8^School of Pharmaceutical Sciences, University Sains Malaysia, George, Malaysia

## Abstract

**Background:**

Medicinal plants have long been used for the treatment of type 2 diabetes mellitus (T2DM). This study aimed to investigate the hypoglycemic efficacy and safety of NW Low-Glu® (contents of one capsule are 300 mg Mas Cotek + 100 mg *Cinnamomum cassia L*. + 250 mg *Nigella sativa* L. powdered extracts) in treatment-naïve, newly diagnosed T2DM patients.

**Methods:**

This was a 12-week, double-blind, double-dummy, randomized, phase 2 clinical trial. A total of 232 male and female patients aged ≥18 and ≤65 years who were newly diagnosed with T2DM and have not received any antidiabetic drugs before and were equally randomized to receive metformin (2000 mg per day), low-dose NW Low-Glu® (content of four capsules per day), or high-dose NW Low-Glu® (content of five capsules per day). Our primary objective was to measure the mean change in HbA1c between each of the experimental arms and the metformin arm.

**Results:**

There was a significant reduction in mean HbA1c at 12 weeks compared to baseline in the low-dose (0.6 (1.4)%; *p*=0.002) and high-dose arms (0.8 (1.7)%; *p*=0.004). There was also a significant reduction in 2 hr PPG at 12 weeks in the low-dose (35.4 (74.9) mg/dL, *p*=0.001) and high-dose arms (24.7 (100.8) mg/dL, *p*=0.04). Weight reduction was significantly higher with both high-dose (1.1 (−1.7) Kg; *p*=0.005) and low-dose arms (0.9 (−1.5) Kg; *p*=0.023) compared to metformin (0.8 (−1.8) Kg). No serious AEs or deaths were reported.

**Conclusions:**

After 3 months of treatment, NW Low-Glu® was noninferior to metformin in reducing HbA1c and 2 hr PPG, while leading to significantly higher weight reduction in newly diagnosed T2DM patients. It was also safe and well tolerated.

## 1. Introduction

Despite the advancements in drug discovery and the increase in the total budget spent on pharmaceutical research and development, the number of new drug approvals has decreased in the recent years. Not only do plants serve as foods, but they have also been an integral part of therapeutic interventions in traditional and alternative medicine throughout human history. Because plants comprise a large number of monomeric compounds, herbal medicine continues to play a multi-targeted approach role in treating diseases [1]. It is estimated that 25% of modern drugs are derived from natural products. In addition, the World Health Organization (WHO) estimates that between 65% and 80% of developing countries use medicinal plants as remedies [2]. In a study conducted in Alexandria, Egypt, the rate of using complementary and alternative medicine (CAM) at least once among type 2 diabetes mellitus (T2DM) patients was 41.7%, with 26.3% being regular CAM users. The main reason for resorting to CAM was the belief in its benefits [3].

The powdered extracts of *Ficus deltoidea* leaves, *Cinnamomum cassia* bark, and *Nigella Sativa* seeds (Black Seeds) have been used for the treatment of DM and other medical conditions for over 2000 years [4–6]. Studies on the individual effect of each of these medicinal plants have demonstrated antihyperglycemic efficacy in individuals with T2DM. For example, Kalman et al. reported significant reduction in glucose and lipid levels upon testing the antidiabetic effects of F*. deltoidea* in adults with prediabetes [7]. There is also strong evidence to support the efficacy of *Cinnamomum cassia* in lowering fasting plasma glucose (FPG) [8]. In a randomized controlled trial by Mang et al., poorly controlled T2DM patients receiving an aqueous extract equivalent to 3 grams of *Cinnamomum cassia* for 4 months had significantly higher reduction in FPG compared to placebo (10.3% vs 3.4%) [9]. Khan et al. also found statistically significant and clinically relevant improvements in FPG of T2DM patients in their clinical trial [10]. Furthermore, Bamosa et al. found that Nigella Sativa seeds' extract (in combination with oral hypoglycemic drugs) led to significant reductions in FBG, 2-hourpost-prandial glucose (2 hr PPG), HbA1c, and insulin resistance at 12 weeks, concluding that it could possibly be a beneficial adjuvant therapy to oral hypoglycemics in patients with uncontrolled T2DM [11].

After thorough literature search, we did not find any clinical study examining the efficacy and safety of a fixed dose combination of the mentioned medicinal herbs. Accordingly, we attempted to compare the hypoglycemic effect of two doses of an herbal medicinal product—NW Low-Glu® (one capsule constitutes 300 mg Mas Cotek powdered extract [from leaves of *Ficus deltoidea* Jack by aqueous solvent extraction] + 100 mg *Cinnamomum cassia* L. powdered extract +250 mg Black Seed powdered extract [from seeds of *Nigella sativa* L. by 70% hydro-alcoholic extraction]) in patients newly diagnosed with T2DM. NW Low-Glu® is registered as a dietary supplement in the Malaysian market under the registration number MAL15070037 T. Given that it has been marketed for years in Malaysia as a herbal traditional medicine, we preferred to investigate NW Low-Glu® in a phase II clinical trial in agreement with the WHO operational guidance supporting clinical trials of herbal products, which states that “substantial prior human use of traditional dose regimens of herbal medicines generally conveys reasonable confidence that these regimens can safely be administered to small numbers of carefully monitored clinical subjects in Phase II trials” [12].

## 2. Subjects and Methods

### 2.1. Study Population

The study included male and female patients aged ≥ 18 and ≤65 years, newly diagnosed with T2DM [FPG ≥126 mg/dl or 2 hr PPG ≥200 mg/dl during OGTT, and HbA1c ≥ 6.5%] who have not received any previous antidiabetic medications (i.e., treatment-naïve). Patients had to be able and willing to perform self-monitoring of blood glucose (SMBG), complete subject diaries, and provide written informed consent.

Exclusion criteria were pregnant or breast-feeding women (women of childbearing potential must have agreed to use an accepted method of contraception during the study and for 1 month after their last dose of study drugs); patients with BMI >40 Kg/m^2^ or < 18.5 Kg/m^2^; eGFR <60 mL/min/1.73 m^2^ (measured by the CKD-EPI equation); human immunodeficiency virus (HIV) positive patients, those with positive hepatitis B surface antigen (HBsAG) or positive hepatitis C virus (HCV) antibody test; patients with type 1 diabetes mellitus (T1DM), diabetes resulting from pancreatic injury or secondary forms of diabetes such as Cushing's syndrome or acromegaly; patients with a history of diabetic complications such as diabetic ketoacidosis (DKA), lactic acidosis, hyperosmolar hyperglycemia, diabetic proliferative retinopathy, severe diabetic neuropathy (requiring treatment with antidepressants or opioids), or a history of decompensated diabetes (polyuria, polydipsia, nocturia, fatigue); history of chronic gastrointestinal (GI) conditions that could impede gastric emptying or potentially affect interpretation of study data; history of weight loss surgery such as gastric bypass, gastric stapling, or gastric banding; history of an eating disorder (e.g., bulimia, anorexia); history of malignancy (except treated basal or squamous cell skin cancer) within 5 years prior to screening; history of significant cardiovascular diseases (such as congestive heart failure, myocardial infarction, coronary heart disease) or uncontrolled hypertension; history of clinically significant renal or liver disease. In addition, patients who had received an investigational drug within 30 days prior to screening, those who were already enrolled in another investigational trial; patients with known or suspected allergy to the trial's interventions; or patients with any condition that, in the judgment of the investigator, would interfere with their ability to comply with all study requirements or that would place them at unacceptable risk; were also excluded.

## 3. Methods

This was a 12-week, double-blind, double-dummy, randomized, active-controlled, parallel-group, interventional, phase 2 clinical trial to investigate the efficacy and safety of NW Low-Glu® (a herbal medicinal product; one capsule constitutes 300 mg Mas Cotek powdered extract [from leaves of *Ficus deltoidea* Jack by aqueous solvent extraction] + 100 mg *Cinnamomum cassia* L. powdered extract +250 mg black seed powdered extract [from seeds of *Nigella sativa* L. by 70% hydro-alcoholic extraction]) in newly diagnosed T2DM patients. Using Interactive Web Response System (IWRS), eligible patients were randomized in a 1 : 1:1 ratio to receive the active control (metformin 2000 mg per day; arm 1), low-dose NW Low-Glu® (content of four capsules [1200 mg Mas Cotek powdered extract, 400 mg *Cinnamomum cassia* L. powdered extract, and 1000 mg black seed powdered extract]; arm 2), or high-dose NW Low-Glu® (content of five capsules [1500 mg Mas Cotek powdered extract, 500 mg *Cinnamomum cassia* L. powdered extract, and 1250 mg black seed powdered extract]; arm 3). Blinding procedure is explained thoroughly in the Supplementary Appendix. This study was conducted from September 2018 to May 2021 in the National Research Center in Cairo, Menoufia University Hospital, Alexandria University Hospital, Fayoum General Hospital, Assiut General Hospital, and Mansoura University Hospital. It was approved by the institutional review boards of all study centers and the Egyptian Ministry of Health. The study complied with the Declaration of Helsinki and Egypt's laws and regulations. This trial was retrospectively registered on clinicaltrials.gov with a registration number NCT05343767.

### 3.1. Sample Size Calculation

The primary objective of our study was to compare the hypoglycemic efficacy of two doses of NW Low-Glu® to that of metformin, as measured by the mean change in HbA1c levels after three months of treatment in newly diagnosed T2DM patients.

According to the 14-week study by Garber et al. [13], the mean change in HbA1c was -0.9% in patients receiving 1000 mg of metformin twice daily. Assuming that there will be no difference between the control (metformin 2000 mg daily) and experimental treatments (NW Low-Glu®), then a sample of 62 patients per treatment arm was deemed appropriate to be 95% sure that the lower limit of a one-sided 95% confidence interval will be above the non-inferiority limit of 0.9% in HbA1c reduction. Considering a sample power of 80%, a standard deviation of the outcome among the lowest dose of ±2%, and an expected drop-out rate of 10% during the 12 weeks of this study; a sample of 68 patients per each treatment arm was found appropriate.

### 3.2. Study Endpoints

The primary endpoint was the mean change in HbA1c between each experimental arm and the active control arm at 12 weeks. The secondary endpoints were the mean change in FPG, 2 hr PPG, and HOMA-IR; between each experimental arm and the active control arm; at 12 weeks. Additionally, the proportion of patients achieving glycemic control (HbA1c < 7%) and safety endpoints (number, nature, and severity of adverse events (AEs) and their causal relationship to study medications) among treatment arms were among the secondary endpoints. Exploratory endpoints were the mean change in HOMA-β, body weight, and plasma insulin alpha-glucosidase activity levels between each experimental arm and the active control arm.

### 3.3. Statistical Analysis

#### 3.3.1. Analysis Population

Primary analysis was done by an intention-to-treat (ITT) analysis including all eligible enrolled patients with at least one treatment dose and HbA1c assessment who had attended any post-treatment visit. Efficacy variables were analyzed using the last-observation-carried-forward convention (LOCF).

### 3.4. Descriptive Analysis

We summarized normally distributed quantitative data using mean and standard deviation (SD), non-normally distributed quantitative variables using median and interquartile range (IQR), and categorical variables using counts and percentages.

### 3.5. Comparative Analysis

Student's *t*-test and one-way ANOVA were used to estimate the comparison between the treatment arms for numerical variables. Paired *t*-test and repeated measure ANOVA test were used to estimate the change in numerical variables throughout the study visits. For categorical variables, Chi^2^ test was used for unpaired variables and McNemar's test was used for paired variables. All tests were performed on the 5% level of significance. Missing data were not counted in the percentages.

## 4. Results

### 4.1. Study Population

**Figure 1:**
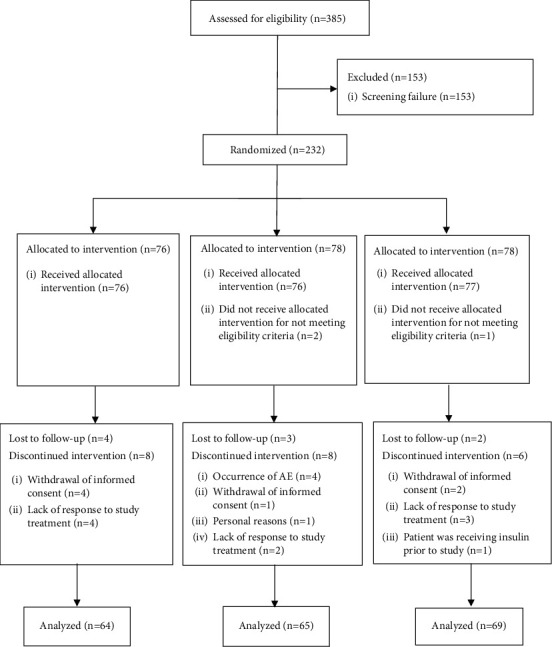
Flow diagram of study patients according to the consolidated standards of reporting trials (CONSORT) for randomized controlled trials.

We enrolled a total of 385 patients in this study; 232 were eligible for randomization (safety population). Of those, 3 patients did not fulfil the eligibility criteria, 22 withdrew from the study, and 9 were lost to follow-up, leaving a total of 198 patients as the efficacy population of the study.  Figure 1 shows the consort flow diagram of the study.

#### 4.1.1. Baseline Characteristics

Most of our patients were never-smokers (163/198; 82.3%) and married (185/198; 93.4%). A large proportion were employed (116/198; 58.6%), with a secondary education (113/198; 57%) and a familial history of diabetes (126/198; 63.6%).  Table 1 shows the demographics and baseline characteristics of the study population.

### 4.2. Primary Endpoint Analysis

The mean HbA1c significantly decreased from 8.5 (1.4)% and 8.8 (1.9)% at baseline to 7.8 (1.6)% and 8.1 (1.8)% after 12 weeks of treatment with a mean reduction of 0.6 (1.4)% (*p*=0.004) and 0.8 (1.7)% (*p*=0.002) for the low-dose arm and the high-dose arm, respectively. On the other hand, the mean HbA1c significantly decreased from 8.7 (1.9)% at baseline to 7.7 (1.6)% (*p*=0.002) after 12 weeks of treatment with metformin. However, there was no statistically significant reduction in HbA1c levels upon comparison of each of the experimental arms to the control arm at week 12. More details are provided in Table 2.

### 4.3. Secondary and Exploratory Endpoint Analyses

There was no significant difference among the mean changes in 2 hr PPG among either of the experimental arms and the active control arm. However, there was a significant reduction in 2 hr PPG between baseline and week 12 in each of the treatment arms (mean changes were −38.2 (101.6) mg/dL, *p*=0.01; −35.4 (74.9) mg/dL, *p*=0.001; and −24.7 (100.8) mg/dL, *p*=0.04 in the metformin, low-dose, and high-dose arms, respectively). Additionally, body weight was significantly reduced in all treatment arms by the end of the study (mean reductions were 0.8 (1.8) kg, *p*=0.001; 0.9 (1.5) kg, *p*=0.001; 1.1 (1.7) kg, *p*=0.001). There was also a significant difference between the mean body weight at the end of the study between patients receiving metformin and those receiving high-dose NW Low-Glu® (*p*=0.005). Moreover, plasma alpha-glucosidase activity was significantly reduced in the high-dose NW Low-Glu® arm (a reduction of 0.4 (1.4); *p*=0.04). On the other hand, there were no significant differences in FPG, HOMA-IR, or HOMA-*β* levels between baseline and the end of the study in any of the treatment arms; nor between any of the NW Low-Glu® arms and the metformin arm at 12 weeks. Further details on secondary endpoints are provided in Table 2.

The proportion of patients achieving target HbA1c (<7%) was similar across the three treatment arms.  Table 3 provides more details.

Ten adverse events (AEs) were experienced by five patients. The prevalence of AEs was 1.3%, 5.1%, and 6.4% in the metformin, low-dose NW Low-Glu®, and high-dose NW Low-Glu® arms, respectively. One event (upper abdominal pain) in the metformin arm was severe and led to patient's withdrawal from the study. No serious adverse events (SAEs) were experienced by any of the study population.  Table 4 provides further details on the safety profile of received interventions.

Binary logistic regression showed that lower 2 hr PPG and HOMA-*β*, as well as shorter duration of diabetes were all significant predictors of achieving target HbA1c (Table 5).

## 5. Discussion

The decoction of boiled leaves of *Ficus deltoidea* Jack has traditionally been used to control blood glucose in the management of diabetes, especially in tropical regions and in the Peninsular Malayasia [7]. The flavonoids vitexin and isovitexin were found to significantly reduce the post-prandial blood glucose level and exhibit in vivo and in vitro alpha-glucosidase activities in preclinical studies [7, 14]. A hot aqueous extract of *Ficus deltoidea* was found to significantly stimulate insulin secretion reaching up to 7.3-folds. Insulin secretion is mediated through the *K* + ATP channel-dependent pathway, which subsequently leads to a rise in the level of intracellular Ca^2+^ in β-pancreatic cells and through a *K* + ATP channel-independent pathway as well (which still remains unclear) [15].

Cinnamon bark has widely been used worldwide as a spice and as a flavoring agent [8]. Its antidiabetic potential was demonstrated in several in vitro and in vivo studies through its insulin sensitizing mechanism [16]. Cinnamon polyphenols are the main constituents responsible for the insulin-like properties of cinnamon; they stimulate glucose uptake in skeletal muscles and adipose tissues [17].

Nigella sativa seeds have been used to prevent and fight several diseases, especially in the Middle East and India. They have hypoglycemic, hypolipidemic, and antioxidant properties [18]. Supplementation with *N. sativa* significantly improves FPG and HbA1c [19].

Given that HbA1c is an integration of both FPG and 2 hr PPG variations over 3 months, the best assessment of glycemic control should optimally be provided by the determination of HbA1c, FPG, and 2 hr PPG [20]. That's why we chose to measure those three variables to determine the level of hypoglycemic efficacy of NW Low-Glu®, with HbA1c reduction being the primary endpoint.

We found that the mean (SD) reduction in HbA1C was 0.9 (1.9) % in patients receiving metformin, 0.6 (1.4)% in the low-dose NW Low-Glu® arm, and 0.8 (1.7)% in the high-doseLow-Glu® arm. There were no statistically significant differences in the mean HbA1c change among these three arms; however, the mean HbA1c at 12 weeks (end of study) was significantly reduced compared to baseline HbA1c in each of the three arms. As per the 2022 clinical practice recommendations of the American Diabetes Association (ADA), metformin is the preferred initial pharmacologic agent for the treatment of T2DM [21]. The fact that NW Low-Glu® demonstrated equivalent HbA1c reduction to metformin in this study is very promising. Although HbA1c change from baseline was significant in all study arms, the mean HbA1c at 12 weeks in all arms remained above the recommended glycemic target—the ADA recommends an HbA1c goal less than 7% if it can be achieved without hypoglycemia or other treatment-related adverse events [22]. At 12 weeks, the mean HbA1c was 7.7 (1.6)%, 7.8 (1.6)%, and 8.1 (1.8)% in the metformin, low-dose NW Low-Glu®, and high-dose NW Low-Glu® arms, respectively. Hence, we expect that the treating physicians of patients involved in this trial have taken it upon themselves so as to optimize the patients' treatment so as to comply with the standard treatment goal. It should be noted though that the percentages of patients achieving HbA1c < 7% in our study were 41.2%, 35.2%, and 26.9% in the metformin, low-dose NW Low-Glu®, and high-dose NW Low-Glu® arms, respectively.

On the other hand, the reduction in the mean FPG was 9.5 (59.1) mg/dL, 13.4 (66.0) mg/dL, and 10.5 (65.2) mg/dL in the metformin, low-dose NW Low-Glu®, and high-dose NW Low-Glu® arms, respectively. Although the Kruskal-Wallis test showed that the three arms are significantly different from each other, differences from baseline were not statistically significant in any treatment arm. Additionally, the mean FPG at 12 weeks was 154.5 (68.5) mg/dL, 144.8 (53.6) mg/dL, and 159.2 (50.4) mg/dL in the metformin, low-dose NW Low-Glu®, and high-dose NW Low-Glu® arms, respectively. Given that the FPG recommendations of the American Association of Clinical Endocrinologists and American College of Endocrinology (AACE/ACE), ADA, and International Diabetes Federation (IDF) are <110 mg/dL [23], 80–130 mg/dL [22], and <100 mg/dL [24], respectively, the FPG levels reached in our study are still beyond the desired targets.

Last in the glucose triad is the 2 hr PPG. We found that the reduction in the mean 2 hr PPG was 38.2 (101.6) mg/dL, 35.4 (74.9) mg/dL, and 24.7 (100.8) mg/dL in the metformin, low-dose NW Low-Glu®, and high-dose NW Low-Glu® arms, respectively. By the end of the study, 2 hr PPG levels ([200.6 (88.5) mg/dL, 198.5 (77.5) mg/dL, and 221.7 (84.8) mg/dL in the metformin, low-dose NW Low-Glu®, and high-dose NW Low-Glu® arms, respectively] were all significantly reduced compared to their baseline levels. Nevertheless, these levels still lie above the recommended targets by the AACE/ACE (<140 mg/dL) [23] and the ADA (<180 mg/dL) [22].

Alpha-glucosidase degrades simple monosaccharides to glucose. As a result, the inhibition of alpha-glucosidase can suppress carbohydrate digestion, delay glucose uptake, and reduce blood glucose levels [25]. As a result, the inhibition of alpha-glucosidase activity can result in a substantial delay in post-prandial hyperglycemia and a favorable impact on insulin resistance and glycemic index regulation [26]. In an in vitro study conducted by Adam et al., hot aqueous, ethanolic, and methanolic extracts of *Ficus deltoidea* were found to significantly inhibit rat intestine alpha-glucosidase activity in a dose-dependent manner [27]. Vitexin and isovitexin were also found to inhibit alpha-glucosidase activity in an in vivo study by Choo et al., as mentioned earlier [4]. Moreover, the freeze-dried*Cinnamon cassia* extract strongly inhibited alpha-glucosidase compared to acarbose in an in vitro study [28]. Similarly, acetone extracts of *Nigella Sativa* had a comparable in vitro inhibitory effect on intestinal alpha-glucosidase to that of acarbose [29]. In our study, high-dose NW Low-Glu® significantly reduced the mean plasma alpha-glucosidase activity from 1.7 (1.5) at baseline to 1.4 (1.3) at 12 weeks, with a mean reduction of 0.4 (1.4). These findings demonstrate one of the possible mechanisms of the action of NW Low-Glu®.

Weight loss in T2DM patients is known to improve glycemic control, reduce the risk of cardiovascular events, and reduce the need for antidiabetic, antihypertensive, and antihyperlipidemic drugs. Metformin is among the weight-neutral antidiabetic drugs and therefore might not be the most suitable drug for an overweight T2DM patient (especially with a sedentary lifestyle) [30]. The fact that both doses of NW Low-Glu® significantly reduced body weight compared to metformin is promising.

When it comes to the factors associated with achieving HbA1c < 7% with NW Low-Glu®, we found that lower baseline 2 hr PPG and HOMA-*β*, and shorter duration of diabetes were all significant factors. This comes in line with the systematic review and meta-analysis conducted by Ketema et al., which found that a 2 hr PPG was closely associated with HbA1c [31]. Hershon et al. described PPG as a “significant contributor to HbA1C that is often overlooked” [32]. In addition, elevated HbA1c values (≥7%) were associated with substantial reductions in *β*-cell function [33], which validate our findings. Further, the UK Prospective Diabetes Study (UKPDS) showed that *β*-cell function assessed by homeostasis model assessment (HOMA) in T2DM patients was already reduced by 50% upon diagnosis and it kept declining by 5% on a yearly basis, which means that a shorter duration of diabetes is associated with *β*-cell function preservation [34].

The rate of AEs was 1.3%, 5.1%, and 6.4% in the metformin, low-dose NW Low-Glu®, and high-doseLow-Glu® arms, respectively. In the low-dose arm, the rates of diarrhea, hyperglycemia, and upper abdominal pain were 1.3%, 1.3%, and 2.6%, respectively. In the high-dose arm, the rates of hyperglycemia, ketoacidosis, and headache were 2.6%, 1.3%, and 1.3%, respectively. None of those adverse events were severe, and none were serious.

Our findings were limited by the short duration of the study. It is possible that the reductions in FPG, HOMA-IR, and HOMA-*β* were not significant owing to the limited time of receiving the interventions. Had the study been longer, we could have determined if the efficacy of NW Low-Glu® is sustainable and whether reaching the recommended targets of the glycemic triad was possible with NW Low-Glu®. In addition, the factor of lifestyle modifications (such as diet and exercises) was not unified across all the patients. Hence, reductions in endpoints should be considered in that light.

## 6. Conclusions

In conclusion, the results of our study revealed that NW Low-Glu® was non-inferior to metformin in reducing HbA1c, FPG, and 2 hr PPG in patients with T2DM after 3 months of treatment. On the other hand, it led to a significantly higher weight reduction compared to metformin. It was also safe and well tolerated. NW Low-Glu® could be a safe and effective hypoglycemic option in the newly diagnosed T2DM patients. It may also be a promising option in people with prediabetes. Yet, none of the patients in any of the treatment arms reached the internationally recommended targets for the glycemic triad.

Based on our results, we believe that NW Low-Glu® should be employed in further investigations as a part of a phase 3 clinical study that should be planned to last for 6 months at least. The phase 3 study should include treatment-naïve prediabetic and newly diagnosed patients with T2DM while incorporating lifestyle and diet modifications—a factor that was lacking in this study.

## Figures and Tables

**Table 1:** Demographics and baseline characteristics of the study population (N = 198).

	Metformin Arm 1 (*N* = 64)	Low-dose NW Low-Glu® Arm 2 (*N* = 65)	High-dose NW Low-Glu® Arm 3 (*N* = 69)
*Characteristic*			
Age (yrs), *median (IQR)*	49 (16)	50 (14)	39 (13.5)
BMI (kg/m^2^), *mean* *±* *SD*	33.1 ± 3.8	31.5 ± 3.9	31.6 ± 4.2
SBP (mmHg), *mean* *±* *SD*	124.6 ± 10.4	124.2 ± 13.4	123.1 ± 12.02
DBP (mmHg), *mean* *±* *SD*	78.9 ± 6.2	77.9 ± 7.9	77.4 ± 6.9
Diabetes duration (months), *mean* *±* *SD*	7.1 ± 9.9	6.3 ± 9.5	6.8 ± 10.1
Smoking duration (yrs), *mean* *±* *SD*	18.5 ± 8.7	21.8 ± 12.1	25.2 ± 9.6
*Gender, no. (%)*			
Females	41 (64.1)	33 (50.8)	37 (53.8)
Males	23 (35.9)	32 (49.2)	32 (46.4)

Marital status, *no. (%)*			
Single	0	2 (3.1)	0
Married	59 (92.2)	60 (92.3)	66 (95.7)
Widow	3 (4.7)	2 (3.1)	2 (2.9)
Divorced	2 (3.1)	1 (1.5)	1 (1.4)

Education, *no. (%)*			
None	7 (10.9)	4 (6.2)	1 (1.4)
Basic/primary	11 (17.2)	8 (12.3)	14 (20.3)
Secondary	37 (57.8)	35 (53.8)	41 (59.4)
Graduate or higher	9 (14.1)	18 (27.7)	13 (18.8)

*Employment, no. (%)*			
Unemployed	25 (39.1)	21 (32.3)	23 (33.3)
Employed	33 (51.6)	39 (60.0)	44 (63.8)
Retired	6 (9.4)	5 (7.7)	2 (2.9)

Residence, *no. (%)*			
Urban	56 (87.5)	59 (90.8)	62 (89.9)
Rural	8 (12.5)	6 (9.2)	7 (10.1)

*Physical examination, no. (%)*			
Normal	64 (100)	65 (100)	69 (100)
Abnormal	0	0	0

*Family history of diabetes*			
*no. (%)*	41 (64.1)	40 (61.5)	45 (65.2)

Smoking habits*, no. (%)*			
Never	53 (82.8)	52 (80.0)	58 (84.1)
Former	3 (4.7)	3 (4.6)	0
Current	8 (12.5)	10 (15.4)	11 (15.9)

**Table 2:** Efficacy variables among treatment arms.

	Metformin Arm 1 (N=64)		Low-dose NW Low-Glu® Arm 2 (N = 65)		High-doseLow-Glu®Arm 3 (N = 69)	
	Baseline	Week 12	Baseline	Week 12	Baseline	Week 12
HbA1c (%)						
Count	64	51	65	54	69	52
Mean (SD)	8.7 (1.9)	7.7 (1.6)	8.5 (1.4)	7.8 (1.6)	8.8 (1.9)	8.1 (1.8)
Mean change (SD)	−0.9 (1.9)		−0.6 (1.4)		−0.8 (1.7)	
*P*-value (Wilcoxon signed rank test)	0.002		0.004		0.002	
Mann–Whitney test comparing Active Control and Arm 2 revealed *p*=0.57; Mann–Whitney comparing Active Control and Arm 3 revealed *p*=0.15; Kruskal–Wallis One-Way ANOVA test comparing the three arms revealed; *p*=0.26						
Fasting plasma glucose (FPG) (mg/dL)						
Count	64	62	65	60	69	63
Mean (SD)	162.9 (62.6)	154.5 (68.5)	158.4 (72.7)	144.8 (53.6)	169.5 (71.7)	159.2 (50.4)
Mean change (SD)	−9.5 (59.1)		−13.4 (66.0)		−10.5 (65.2)	
P value (Wilcoxon signed rank test)	0.2		0.1		0.2	

Mann–Whitney test comparing Active Control and Arm 2 revealed *p*=0.414; Mann–Whitney test comparing Active Control and Arm 3 revealed *p*=0.18; Kruskal–Wallis One-Way ANOVA test comparing the three arms revealed *p*=0.18						
2hr post prandial glucose (2hr PPG) (mg/dL)						
Count	60	60	63	58	67	62
Mean (SD)	237.8 (88.7)	200.6 (88.5)	243.7 (104.3)	198.5 (77.5)	246.2 (88.8)	221.7 (84.8)
Mean change (SD)	−38.2 (101.6)		−35.4 (74.9)		−24.7 (100.8)	
P value (Wilcoxon signed rank test)	0.01		0.001		0.04	

Mann–Whitney test comparing Active Control and Arm 2 revealed *p*=0.9; Mann–Whitney comparing Active Control and Arm 3 revealed *p*=0.1; Kruskal–Wallis One-Way ANOVA test comparing the three arms revealed *p*=0.14						
HOMA-IR						
Count	58	52	59	55	67	52
Mean (SD)	2.4 (1.5)	2.6 (1.7)	2.3 (1.3)	2.1 (1.3)	2.7 (1.8)	2.7 (1.8)
Mean change (SD)	−0.001 (1.3)		−0.3 (1.3)		−0.01± 1.4	
*P*-value (Wilcoxon signed rank test)	0.9		0.1		0.6	

Mann–Whitney test comparing active Control and Arm 2 revealed *p*=0.9; Mann–Whitney test comparing active Control and Arm 3 revealed *p*=0.87; Kruskal–Wallis One-Way ANOVA test comparing the three arms revealed *p*=0.129						
HOMA-*β*						
Count	58	51	58	53	67	52
Mean (SD)	74.3 (49.1)	82.8 (60.6)	77.7 (52.6)	79.7 (49.7)	73.4 (60.7)	77.8 (64.8)
Mean change (SD)	−4.4 ± 68.6		1.2 ±49.1		2.79 ± 78.7	
*P* value (Wilcoxon signed rank test)	0.9		0.4		0.4	

Mann–Whitney test comparing Active Control and Arm 2 revealed *p*=0.86; Mann–Whitney test comparing Active Control and Arm 3 revealed *p*=0.43; Kruskal–Wallis One-Way ANOVA comparing the three arms revealed; *p*=0.62						
Weight (Kg)						
Mean (SD)	91.2 (10.6)	90.4 (10.9)	88.4 (12.5)	87.5 (12.8)	85.6 (12.5)	84.5 (12.2)
Mean change (SD)	−0.8 ±1.8		−0.9 ± 1.5		−1.1 ± 1.7	
*P* value (Wilcoxon signed rank test)	0.001		0.001		0.001	

Mann–Whitney test comparing Active Control and Arm 2 revealed *p*=0.14; Mann–Whitney comparing Active Control and Arm 3 revealed *p*=0.005; Kruskal–Wallis One-Way ANOVA comparing the three arms revealed *p*=0.023						
Plasma alpha-glucosidase activity (nmol/mL/hour)						
Count	60	62	63	60	67	65
Mean (SD)	1.6 (1.3)	1.6 (1.3)	1.5 (1.1)	1.6 (1.3)	1.7 (1.5)	1.4 (1.3)
Mean change (SD)	−0.04 ± 1.9		0.12 ± 1.7		−0.4 ± 1.4	
*P* value (Wilcoxon signed rank test)	0.23		0.5		0.04	
Mann–Whitney test comparing Active Control and Arm 2 revealed *p*=0.867; Mann–Whitney test comparing Active Control and Arm 3 revealed *p*=0.42; Kruskal–Wallis One-Way ANOVA test comparing the three arms revealed; *p*=0.59						

**Table 3:** Proportion of patients achieving target HbA1c < 7% in each treatment arm.

	Metformin Arm 1 (*N* = 51)		Low-dose NW Low-Glu®Arm 2 (*N* = 54)		High-dose NW Low-Glu® Arm 3 (*N* = 52)	
Count (%)	95% CI	Count (%)	95% CI	Count (%)	95% CI
HbA1c < 7%	21 (41.2)	27.2–55.2	19 (35.2)	22.0–48.3	14 (26.9)	14.5–39.4
HbA1c ≥ 7%	30 (58.8)	44.8–72.8	35 (64.8)	51.7–77.9	38 (73.1)	60.6–85.6

Chi-square test used to compare between three arms revealed *p*=0.31. *Note.* Percentages were calculated from patients with available HbA1c measurements at week 12.

**Table 4:** Incidence rate of AEs and their specifications and characteristics.

	Metformin Arm 1 (*N* = 76)	Low-dose NW Low-Glu® Arm 2 (*N* = 78)	High-dose NW Low-Glu® Arm 3 (*N* = 78)	Total (*N* = 232)
Frequency of AEs, *no. of events (%)*	1 (1.3)	4 (5.1)	5 (6.4)	10 (4.3)
*AE specification*				
Diarrhea	0	1 (1.3)	0	1 (0.4)
Upper abdominal pain	1 (1.3)	2 (2.6)	0	3 (1.3)
Hyperglycemia	0	1 (1.3)	2 (2.6)	3 (1.3)
Ketoacidosis	0	0	1 (1.3)	1 (0.4)
Headache	0	0	1 (1.3)	1 (0.4)
Somnolence	0	0	1 (1.3)	1 (0.4)

*Characteristics of AEs*				
Intensity				
Mild	0	3 (3.8%)	0	3 (30)
Moderate	0	1 (1.3%)	5 (6.4%)	6 (60)
Severe	1 (1.3%)	0	0	1 (10)

*Relatedness to study medication*				
Related	0	0	0	0
Probably/Likely related	1 (1.3%)	0	0	1 (10)
Possibly related	0	0	5 (6.4%)	5 (50)
Unlikely related or probably not related	0	1 (1.3%)	0	1 (10)
Unrelated	0	3 (3.8%)	0	3 (30)

*Seriousness criteria*				
Serious	0	0	0	0
Not serious	1 (100%)	4 (100%)	5 (100%)	10 (100)

**Table 5:** Binary logistic regression analysis of diabetic patients achieving target HbA1c < 7%.

Variables	B	Wald.	*p* value	OR	95% CI	
					Lower	Upper
Baseline HbA1c (%)	−0.203	1.3	0.2	0.817	0.579	1.151
Fasting plasma glucose (mg/dL)	0.007	1.2	0.2	1.007	0.995	1.019
2h-post prandial glucose (mg/dL)	−0.010	7.02	0.008	0.990	0.983	0.997
HOMA-*β*	0.011	5.5	0.02	1.011	1.002	1.020
Diabetes duration (months)	0.094−	11.4	0.001	0.910	0.862	0.961
Patients' weight (Kg)	0.024	1.5	0.2	1.024	0.987	1.063

The logistic regression model was statistically significant, revealed *p*=0.001; the model showed prediction accuracy with 75.7%.

## Data Availability

The data supporting the findings of this study are available upon request from the corresponding author.

## Supplementary Materials

Supplementary Materials The procedure for blinding used in the study is supplied in Supplementary Appendix 1.
