# Supplementary material for: A Phase II, Randomized, Double-Blind, Double-Dummy, Active-Controlled Clinical Trial to Investigate the Efficacy and Safety of NW Low-Glu® in Patients Newly Diagnosed with Type 2 Diabetes Mellitus
Source: Evid Based Complement Alternat Med. 2022 Sep 27;2022:9176026. doi: 10.1155/2022/9176026 (PMC9532066; doi:10.1155/2022/9176026)
Supplement: Supplementary Materials — The procedure for blinding used in the study is supplied in Supplementary Appendix 1. [file 9176026.f1.docx]

**Blinding procedure**

Investigational product was packed in HDPE (High-density polyethylene) white bottles. Bottles were packed in white carton boxes. A label identified the Sponsor’s name, study name, site number, patient’s initials, randomization number, visit number, visit date (dispensation date), batch number, number of capsules/tablets, expiry date, storage instructions and instructions to keep out of the reach of children in addition to a statement that this medication is for clinical trial use only. Investigational product was stored at ambient temperature (not exceeding 30°c). During each study visit (from baseline visit to visit 5), each patient received a number of bottles containing the amount of medications sufficient for 2-4 weeks (according to visit interval). Tablets containing metformin and those containing placebo were identical in physical appearance and mode of administration. Capsules containing NW Low-Glu and those containing placebo were identical in physical appearance and mode of administration.

**Dose selection of the two doses of NW Low-Glu^®^ in the study was based on the findings of preclinical (pharmacological and toxicological) studies performed on Sprague Dawley rats:**

1. **Pharmacology study:**

- Effect of NW-Low-Glu formula in a rat model of type-II diabetes was examined.
- Diabetes was induced by intraperitoneal injection of a freshly prepared solution of streptozotocin (STZ, 45 mg/kg) in 0.1M citrate buffer, pH (4.5) in overnight fasted rats. After 48 h of STZ administration, fasting blood glucose (FBG) levels was measured. Rats having FBG values > 200-300 mg/dl will be considered diabetic and employed for the screening and assays. Treatment was carried out for 4 weeks as follows: the first and the second groups received only the vehicle (distilled water) orally and served as the normal and diabetic control groups; respectively. The 3rd, 4th and 5th groups were received NW Low-Glu (125, 250 and 500 mg/kg/day) orally. The last group was orally administered metformin (150 mg/kg/day) as reference control groups.
- In summary, treatment was carried out as follows:
  - The **normal control group** received only the vehicle (distilled water)
  - The **diabetic control group** received only the vehicle (distilled water)
  - The **3rd, 4th and 5th groups** received NW-Low Glu (125, 250 and 500 mg/kg/day)
  - The **reference control group** orally administered metformin (150 mg/kg/day)
- **Results:**

| **Groups** | **Fasting Blood glucose (mg/dl)** | | | **Insulin (mIU/ml) at 4 weeks** | **Hb (g/dl) at 4 weeks** | **HbA1c (%) at 4 weeks** |
| --- | --- | --- | --- | --- | --- | --- |
|  | **Baseline** | **2 weeks** | **4 weeks** |  |  |  |
| **Normal Control** | 96.5±2.74ǂ | 93.0±4.66ǂ | 79.5±4.40ǂ | 6.3±0.59ǂ | 14.2±0.38ǂ | 4.9±0.21ǂ |
| **Diabetic control** | 262.1±21.37* | 203.0±3.96* | 208.9±2.08* | 18.9±1.56* | 10.5±0.33* | 9.1±0.38* |
| **NW Low-Glu, 500 mg/kg** | 263.2±22.81* | 94.3±2.64ǂ | 80.0±3.81ǂ | 11.7±0.57*ǂ | 14.5±0.21ǂ | 5.9±0.34ǂ |
| **NW Low-Glu, 250 mg/kg** | 275.0±20.55* | 92.6±2.48ǂ | 84.7±1.33ǂ | 13.6±0.86*ǂ | 12.8±0.23ǂ | 5.9±0.10ǂ |
| **NW Low-Glu, 125 mg/kg** | 263.0±25.83* | 93.0±1.93ǂ | 82.8±2.57ǂ | 14.8±1.65*ǂ | 13.1±0.17ǂ | 5.6±0.19ǂ |
| **Metformin, 150 mg/kg** | 254.7±24.89* | 164.7±16.80*ǂ | 82.0±2.77ǂ | 9.4±0.67ǂ | 13.5±0.32ǂ |  |

Adult male Sprague-Dawley rats received either citrate buffer (normal control) or streptozotocin (45 mg/kg) in citrate buffer (diabetic control) by intraperitoneal injection. Diabetic rats received NW Low Glu (500, 250 or 125 mg/kg, p.o), Metformin (MET, 150 mg/kg, p.o) for four weeks, 48-hours after induction of diabetes.

Data was expressed as mean of 7 experiments ± SEM. Data analysis was done by one way ANOVA followed by Tukey’s multiple comparison post hoc test at *p*≤ 0.05.

* Significantly different from normal control at *p* ≤ 0.05.

ǂ significantly different from diabetic control at *p* ≤ 0.05.

**Conclusion of pharmacology study:** NW- Low Glu significantly reduced FBG, insulin, and HbA1c levels in diabetic Sprague-Dawley rats compared to diabetic controls receiving no treatment.

1. **Toxicity Studies:**

The following table summarizes the findings of all performed toxicity studies, which revealed that the lowest-observed-adverse-effect level (LOAEL) dose is 250 mg/kg.

| **Study** | **Doses** | **Conclusion** |
| --- | --- | --- |
| Single Dose (Acute) | 0 and 2000 mg/kg | Safe |
| Sub-Acute/ 28 days | 0,  250,  500,  1000 mg/kg | 1000 mg/kg NOAEL |
| Sub-Chronic/ 90 days |  | 500 mg/kg NOAEL |
| Reproductive Toxicity |  | 250 mg/kg NOAEL |
| Reproductive Developmental Toxicity |  | 250 mg/kg NOAEL |
| Prenatal Developmental Toxicity |  | 250 mg/kg NOAEL |
| Genotoxicity |  | No toxicity |
| Chronic Toxicity |  | 500 mg/kg NOAEL |

**Conclusion of Preclinical studies:**

The no-observed-adverse-effect level (NOAEL) of NW- Low Glu is **250** mg/kg in 3 of the studies and **500** in 2 of them.

**Conversion of doses revealed from preclinical studies into human doses:**

According to “Guidance for Industry Estimating the Maximum Safe Starting Dose in Initial Clinical Trials for Therapeutics in Adult Healthy Volunteers FDA”^[[1]](#footnote-1)^, the following equation applies:

- **In case of choosing NOAEL to be 250 mg/kg:**

HED (human equivalent dose) = animal dose in mg/kg × 0.16 (or ÷ 6.2)

HED = 250 × 0.16 = 40 mg/kg

Total daily human dosage according to average body weight (60 kg) is:

40 mg × 60 kg = **2400 mg** of NW- Low Glu per day.

- **In case of choosing NOAEL to be 500 mg/kg:**

HED (human equivalent dose) = animal dose in mg/kg × 0.16 (or ÷ 6.2)

HED = 500 × 0.16 = 80 mg/kg

Total daily human dosage according to average body weight (60 kg) is:

80 mg × 60 kg = **4800 mg** of NW- Low Glu per day.

**Conclusion:** 4800 mg per day is the MSRD (Maximum Safe Recommended Dose), but it’s better to apply a safety factor for protection of human subjects receiving the initial clinical dose. This can be accommodated by lowering the human starting dose from the HED of the selected species NOAEL. So, in order to comply with the safety margin, we chose the maximum daily human dose in the range of NOAEL 250 & 500 mg/kg to be 2600 and 3250 mg per day.

1. https://www.fda.gov/regulatory-information/search-fda-guidance-documents/estimating-maximum-safe-starting-dose-initial-clinical-trials-therapeutics-adult-healthy-volunteers [↑](#footnote-ref-1)
